# Characteristics of Suppressor Macrophages Induced by Mycobacterial and Protozoal Infections in relation to Alternatively Activated M2 Macrophages

**DOI:** 10.1155/2012/635451

**Published:** 2012-05-15

**Authors:** Haruaki Tomioka, Yutaka Tatano, Win Win Maw, Chiaki Sano, Yuichi Kanehiro, Toshiaki Shimizu

**Affiliations:** ^1^Department of Microbiology and Immunology, Shimane University School of Medicine, Izumo, Shimane 693-8501, Japan; ^2^Department of Nutritional Sciences, Faculty of Home Economics, Yasuda Women's University, Hiroshima 731-0153, Japan

## Abstract

In the advanced stages of mycobacterial infections, host immune systems tend to change from a Th1-type to Th2-type immune response, resulting in the abrogation of Th1 cell- and macrophage-mediated antimicrobial host protective immunity. Notably, this type of immune conversion is occasionally associated with the generation of certain types of suppressor macrophage populations. During the course of *Mycobacterium tuberculosis* (MTB) and *Mycobacterium avium-intracellulare* complex (MAC) infections, the generation of macrophages which possess strong suppressor activity against host T- and B-cell functions is frequently encountered. This paper describes the immunological properties of M1- and M2-type macrophages generated in tumor-bearing animals and those generated in hosts with certain microbial infections. In addition, this paper highlights the immunological and molecular biological characteristics of suppressor macrophages generated in hosts with mycobacterial infections, especially MAC infection.

## 1. Introduction

Worldwide, tuberculosis (TB) is a major global health concern because it is a highly contagious and life-threatening infection [1–3]. Moreover, the enhanced susceptibility to TB in human immunodeficiency virus- (HIV-) infected populations is another serious health problem [4]. Notably, multidrug-resistant- (MDR-) TB including extensively drug-resistant- (XDR-) TB, is currently increasing in the world [5, 6]. On the other hand, *Mycobacterium avium-intracellulare* complex (MAC) infections are frequently encountered in immunocompromised hosts, especially AIDS patients [7, 8], although nodular-bronchiectasis type MAC infections without predisposing conditions are steadily increasing, particularly in Japan [9, 10].

**Figure 1:**
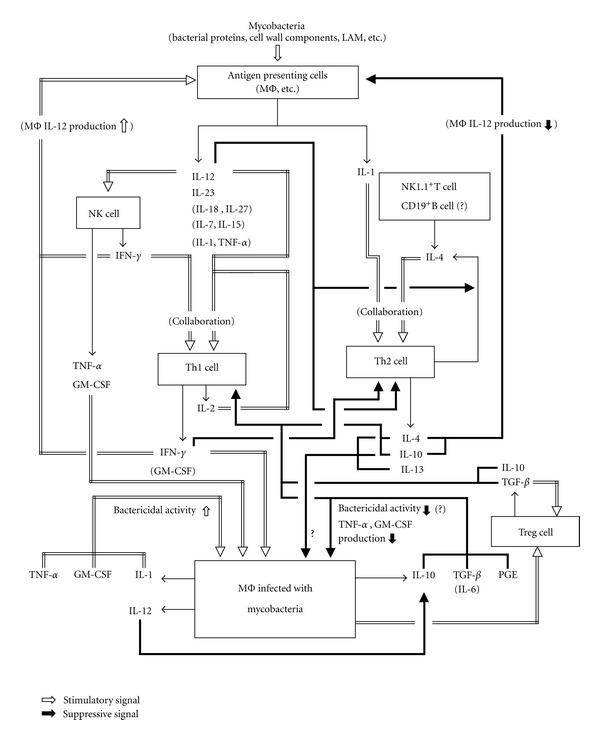
Cytokine networks in hosts with mycobacterial infection. In this cytokine network, proinflammatory cytokines, including IL-12, IL-23, IL-18, and tumor necrosis factor-*α* (TNF-*α*), which are produced by infected macrophages (MΦs) and dendritic cells (DCs), induce the cellular expansion and differentiation of Th1 cells, resulting in enhanced production of Th1 and Th1-like cytokines, such as interferon-*γ* (IFN-*γ*), IL-2, TNF-*α*, and granulocyte-macrophage colony-stimulating factor (GM-CSF). These cytokines play crucial roles in the expression of host resistance against mycobacterial infections. In addition, immunosuppressive cytokines and humoral factors, such as IL-4, IL-10, transforming growth factor-*β* (TGF-*β*), and prostaglandin E (PGE), which are produced by Th2 cells, Treg cells, Th3 cells, and macrophages, appear to play important roles in the establishment of immunodeficiency frequently encountered in persistent and advanced infection with mycobacterial pathogens, including MTB.

 In general, during the early to middle stages of mycobacterial infections, Th1 cell-mediated immune responses are dominant and play crucial roles in the establishment and expression of antimycobacterial host resistance (Figure 1) [11, 12]. However, in the advanced stages of mycobacterial infections such as TB and *M. avium* infection, host immune systems tend to adopt a Th2-type immune response through the induction and activation of Th2 cells, thereby resulting in a diminishment of Th1 cell- and activated macrophage-mediated antimycobacterial cellular immunity (Figure 1) [13–17]. Notably, this type of immune deviation is occasionally associated with the generation of certain types of immunosuppressive macrophage populations. Indeed, during the course of infections with *Mycobacterium tuberculosis* (MTB) and MAC in humans and experimental animals, the generation of macrophage populations that possess strong suppressor activity against host T-cell function is generally observed. It appears that immunosuppressive macrophages, particularly those exerting suppressor activity against T cells, play important roles in mycobacterial persistency in hosts and the establishment of immune unresponsiveness in advanced stages of infection. Therefore, it is important to elucidate the precise nature of such immunosuppressive macrophage populations.

 In this context, we should note the phenomenon of macrophage polarization in bacterial infections, particularly those due to facultative intracellular pathogens, such as mycobacteria, *Salmonella* species, and *Listeria monocytogenes* [17, 18]. Recent studies on the gene expression profiling of macrophages have revealed that various bacteria induce the transcriptional activity of a common host response, which includes genes belonging to the M1 program, associated with macrophage polarization yielding classically activated macrophages (called M1 macrophages) exerting proinflammatory and/or microbicidal functions. However, excessive or prolonged M1 polarization of macrophages leads to tissue injury and contributes to pathogenesis [19]. The so-called alternatively activated macrophages (called M2 macrophages) having immunosuppressive and tissue-repairing functions play critical roles in the resolution of harmful inflammation by producing anti-inflammatory mediators [17–19].

 In this paper article, with the M1 and M2 polarization of macrophages in mind, we will describe the immunological properties of (1) alveolar macrophages which have spontaneous immunosuppressive activity and (2) suppressor macrophages produced in hosts with protozoal infections, and (3) the immunological and molecular biological characteristics of immunosuppressive/suppressor macrophages generated in hosts with mycobacterial infections, especially MAC infection.

## 2. Macrophage Polarization and Suppressor Macrophages

Immunosuppressive/suppressor macrophages induced by microbial infections, including mycobacteriosis and protozoiasis described below, have properties in common with those of M2 alternatively activated macrophages. Thus, this section will deal with the relationship between *in vivo* generation of suppressor macrophages and macrophage M2 polarization. In response to extracellular signals of cytokines and microbial stimuli, cells belonging to the macrophage lineage express specialized and polarized functional properties [18, 20–25]. There are mainly two types of polarized macrophages, generally called M1 and M2 macrophages (Table 1), although some investigators argue against such a classification, because these cells might be able to change from one phenotype to another, and there is no straightforward correspondence of phenotype between T cell subsets and subpopulations of other immune cells [20]. These investigators prefer to call M1 and M2 macrophages “classically activated macrophages” and “alternatively activated macrophages,” respectively. As shown in Table 1, M1 classically activated macrophages are induced to develop by interferon-*γ* (IFN-*γ*) alone or in combination with other macrophage-activating cytokines, including tumor necrosis factor-*α* (TNF-*α*) and granulocyte-macrophage colony-stimulating factor (GM-CSF), and certain microbial stimuli such as lipopolysaccharide (LPS). In contrast, Th2-derived cytokines, IL-4 and IL-13, have been demonstrated to generate M2 alternatively activated macrophages [20, 26, 27]. In this context, it is noteworthy that the M2 alternatively activated macrophages consist of three subpopulations; M2a macrophages induced with IL-4 and IL-13 (classical activation); M2b macrophages (corresponding to Type II-activated macrophages) induced with immune complex and Toll-like receptor (TLR)/IL-1 receptor (IL-1R) ligands via Fc receptor 1 (FcRl), complement receptors and TLR; M2c macrophages generated in response to IL-10 and glucocorticoid hormones [17, 18, 21, 28–30]. Notably, in classical activation of macrophages causing M1 polarization, NF-*κ*B pathway plays a central role in the response to proinflammatory cytokines, such as IFN-*γ*, and microbial-associated molecular patterns [31]. In addition, transcription factor, interferon regulatory factor 5 (IRF5), has recently been reported to act as another important M1 regulatory factor [32]. IRF5 participates in the activation of genes encoding IL-12, IL-23, and proinflammatory cytokines and represses the gene encoding IL-10, resulting activation of macrophages into M1 cells, which are capable of setting up the environment for a potent Th1-Th17 response [32]. Thus, IRF5 functions as a factor, which promotes M1 macrophage polarization. On the other hand, c-Maf, a basic leucine zipper transcription factor, and galectin-3, a carbohydrate-binding lectin expressed on macrophage cell membrane, play crucial roles in M2 polarization, especially in the case of M2 alternatively activated macrophages [33, 34]. Moreover, I*κ*B kinase *β* (IKK*β*) inhibits the M1 classically activated macrophage phenotype through negative cross-talk with the signal transducer and activator of transcription (STAT) 1 pathway [31]. In relation to M1 and M2 polarization, human monocytes induced to differentiate with GM-CSF or macrophage colony-stimulating factor (M-CSF) are also known to have M1 and M2 properties, respectively, and have been called M*ϕ*1 and M*ϕ*2 [35].

 In general, as reported by Martinez et al. [36], M1 and M2 macrophage populations have distinct phenotypes due to differential profiles of gene expression with each other, as follows (Table 1). First, typical M1 classically activated macrophages have a phenotype with a high level production of IL-12 and IL-23 but a low level expression of IL-10. They are efficient producers of cytotoxic effector molecules, such as reactive oxygen intermediates (ROIs) and reactive nitrogen intermediates (RNIs) and inflammatory cytokines, including IL-1*β*, TNF-*α*, and IL-6. Thus, M1 macrophages participate as inducer and effector cells in polarized Th1 responses and mediate resistance against intracellular parasites and tumors [20–22]. In contrast, the various forms of M2 macrophages share a phenotype with a low level production of IL-12 and IL-23 but a high level expression of IL-10. In general, M2 alternatively activated macrophages are characterized by low production of proinflammatory cytokines including IL-1, TNF-*α*, and IL-6. However, M2b macrophages (type II-activated/regulatory macrophages), which are characterized by high levels of IL-10 and CD86 expression, but low levels of IL-12 and arginase 1 expression, are good producer of IL-1, TNF-*α*, and IL-6, as in the case of M1 classically activated macrophages [28–30]. In addition, M2b type II-activated macrophages retain high level expression of inducible nitric oxide synthase (iNOS) and RNI production [28–30].

 Next, M2 macrophages generally have high levels of scavenger, mannose, and galactose-type receptors. Moreover, in M2 macrophages, arginine metabolism is shifted to production of ornithine and polyamines via arginase 1 (Arg 1) [20, 21, 39]. Moreover, M1 classically activated macrophages and the various forms of M2 alternatively activated macrophages have distinct chemokine and chemokine receptor repertoires [21]. M2 macrophages principally play important roles in polarized Th2 reactions. For instance, (1) M2 macrophages promote the killing and encapsulation of parasites; (2) M2 macrophages present in established tumors and promote progression, tissue repair, and remodeling; (3) M2 macrophages have immunoregulatory and anti-inflammatory functions [20, 22]. In addition, it has been indicated that M2 macrophages inhibited the generation of M1 macrophages and that CCL17 and IL-10 mediated the actions of M2 macrophages as humoral effectors [22].

 As clearly described by Muray and Wynn [19], after infection or tissue injury, the first responder macrophages usually exhibit an inflammatory phenotype and secrete proinflammatory mediators, such as TNF-*α*, IL-1, RNIs, and ROIs, thereby causing activation of antimicrobial mechanisms characteristic of M1 classically activated macrophages. These macrophages also generate IL-12 and IL-23, which are decisive in cell expansion and the differentiation of Th1 and Th17 cells [40, 41]. Thus, the M1 program of macrophages is usually associated with protection during acute infectious diseases. However, RNIs and ROIs produced by such activated macrophage populations are toxic and highly damaging to neighbouring tissues. Therefore, antimicrobial/inflammatory M1 classically activated macrophages must be controlled to prevent collateral extensive tissue damage by regulatory mechanisms, including the generation of M2 alternatively activated macrophages, which antagonize M1 polarization of macrophages and also exert strong anti-inflammatory activity. In addition, M2 alternatively activated macrophages play important roles in wound healing and fibrosis by generating growth factors, including transforming growth factor-*β* (TGF-*β*) and platelet-derived growth factor, which act on fibroblasts, epithelial cells, and endothelial cells, causing enhanced cellular growth, angioneogenesis, and the production of extracellular matrix [19, 42, 43].Should we change the highlighted “angioneogenesis” to “angiogenesis” As commented by Lugo-Villarino et al. [17], macrophages undergo different programs of activation, rendering them either proinflammatory and microbicidal (M1 macrophages) or immunosuppressants and tissue repairers (M2 macrophages). An excess of prolonged polarization of either program may be detrimental to the host due to potential tissue injury or contribution to pathogenesis. Indeed, the predominant type 2 inflammatory environment shifts back to type 1 after successful treatment of pulmonary TB in infected patients [15, 17].

 In this context, it has been demonstrated that the common response of macrophages to bacterial infections induced by MTB, *Mycobacterium bovis* BCG, *Bordetella pertussis*, *Chlamydophila pneumoniae*, *Legionella pneumophila*, and so forth, involves upregulation of genes involved in M1 polarization of macrophages. These induced genes encoding cytokines such as TNF, IL-6, IL-12, IL-1*β*, cytokine receptors such as IL-7R, IL-15 receptor *α* (IL-15RA), chemokines such as CCL2, CCL5, and CXCL8, and the chemokine receptor CCR7. On the other hand, IL-1 receptor antagonist (IL-1ra) appears to be the only gene associated with M2 polarization of macrophages that is expressed after bacterial challenge [18, 44]. Some bacterial pathogens have evolved sophisticated strategies to prevent M1 polarization, neutralize microbicidal effectors of macrophages, or promote M2 polarization [18]. With respect to mycobacterial diseases, the following profiles are known. First, during the early phase of MTB infection, M1 polarization of host macrophages is evident and this is in agreement with the clinical profiles of patients with active TB. However, a small population of TB patients is known to exhibit M2 polarization, which can be reversed by effective chemotherapy, indicating the role of M2 polarization in the chronic evolution of TB [18, 45]. In this context, a recent study by Redente et al. demonstrated the following [46]. In their experimental infection model in mice, MTB resulted in pulmonary inflammation characterized by an influx of macrophages, followed by systemic effects on the bone marrow and other organs. In this infection model, pulmonary IFN-*γ* and IL-4 production coincided with the altered polarization of alveolar macrophages. Soon after MTB infection, IFN-*γ* content in bronchoalveolar lavage fluid (BALF) increased, and bronchoalveolar lavage (BAL) macrophages became M1 classically activated macrophages, as characterized by increased expression of iNOS and production of RNIs. As inflammation progressed in the model, the amount of IFN-*γ* in BALF and iNOS expression by BAL macrophages decreased and, thereafter, the IL-4 content in BALF and arginase 1 expression by macrophages rose, indicating M2 polarization of BAL macrophages. Indeed, at 7 days after infection, BAL macrophages were Arg1^low^iNOS^low^. By day 21, BAL macrophages were Arg1^low^iNOS^high^ (M1-type) and those isolated 35 to 60 days after MTB exposure were Arg1^high^iNOS^low^ (M2-type), thereby indicating a switch from M1 to M2 polarization of macrophages. Notably, in this infection model, macrophages present in MTB-induced granulomas remained M1-polarized [46]. In connection with this finding, Ito et al. [47] revealed that the lungs of TLR9-deficient mice, which were injected intravenously with purified protein derivative (a mixture of MTB antigens) 2 weeks after sensitization with complete Freund's adjuvant containing heat-killed MTB, developed a type 2-like response with significantly larger granulomas and increased accumulation of eosinophils compared to control mouse granulomas. This phenomenon was associated with a selectively abrogated type-1 and enhanced type 2 cytokine profile in the lungs. In this case, macrophages in the lungs of TLR9-deficient mice expressed significantly lower levels of the M1 macrophage marker, iNOS, but higher levels of M2 macrophage markers such as arginase 1 and Fizz 1 (found in inflammatory zone 1). These findings showed that lung macrophages were shifted from M1 to M2 type in TLR9-deficient mice, thereby suggesting that TLR9 plays an important role in maintaining the appropriate phenotype in a Th1 granulomatous response. In relation to this, overexpression of IL-10 is characteristic of lepromatous leprosy in humans and this phenomenon is attributable to M2 polarization of host macrophages [18, 45, 48]. The gene expression profile of lepromatous lesions is enriched for M2 genes, such as CD36, CD163, scavenger receptor-A, and macrophage receptor with collagenase structure (MARCO), when compared with tuberculoid lesions. In contrast, antimicrobial profile based on M1 program dominates in tuberculoid lesions [45, 49].

 With special reference to M2 differentiation of macrophages, the following finding by Liao et al. [50] is noteworthy. They found that Krüppel-like factor 4 (KLF4) functions as a critical regulator of macrophage polarization, that is, KLF4 expression was robustly induced in M2 alternatively activated macrophages and strongly reduced in M1 classically activated macrophages. Mechanistically, KLF4 was found to cooperate with STAT6 to induce an M2 genetic program and inhibit M1 targets via sequestration of coactivators required for NF-*κ*B activation. KLF4-deficient macrophages demonstrated increased expression of proinflammatory genes encoding TNF-*α*, iNOS, cyclooxygenase 2, RANTES, and macrophage chemoattractant protein-1 (MCP-1), decreased expression of prototypical target genes that characterize the M2 phenotype, such as arginase-1 (*Arg1*), mannose receptor (*Mrc1*), resistin-like molecule *α* (*Fizz1*), chitinase-like 3 (*Chi313*), enhanced bactericidal activity against *Escherichia coli*, and altered metabolism. These findings indicate that KLF4 as well as other transcription factors such as galectin 3, a good distinctive descriptor of M2a and M2c macrophages, is a regulator of macrophage M2 polarization [17, 37, 38, 50]. It has also been reported that MTB and its cell wall mannose-capped lipoarabinomannan (Man-LAM) induced the expression of peroxisome proliferator-activated receptor *γ* (PPAR-*γ*) in monocyte-derived macrophages through the mannose receptor-dependent pathway [51]. In this context, PPAR-*γ* is a nuclear factor that is characteristic of M2 alternative activation of macrophages, because of its strong expression by M2 macrophages, and is thought to be critical for intramacrophage survival of infected mycobacteria [45, 48]. Notably, activated PPAR-*γ* promoted IL-8 and cyclooxygenase 2 expression in a mannose receptor-dependent manner [51]. Furthermore, MTB- or Man-LAM-induced PPAR-*γ*-mediated IL-8 response was independent of NF-*κ*B activation and TLR-2 expression. In contrast, infection with attenuated *Mycobacterium bovis* BCG induced less PPAR-*γ* expression and elicited IL-8 production in an NF-*κ*B-independent manner. These findings suggest that PPAR-*γ* functions as one important “molecular switch” in regulating macrophage immune responses to MTB, particularly in M2 polarization.

 On the other hand, recent finding by François et al. [52] is also interesting. They demonstrated that human bone-marrow-derived mesenchymal stromal cells (MSCs) derived from normal adult donors possess immunosuppressive potential. Using MSCs from different donors, they showed variability between donors in their ability to suppress T-cell proliferation induced by anti-CD3 and anti-CD28 antibodies. Notably, in this case, enzymatic activity indoleamine 2,3-dioxygenase (IDO), an IFN-*γ*-inducible intracellular enzyme, of MSCs was the main mechanisms of T cell suppression. Moreover, the enzymatic activity of IDO was partially implicated in the differentiation of blood monocytes into IL-10-secreting M2-type immunosuppressive macrophages. Those monocyte-derived M2 alternatively activated macrophages are in turn implicated in the suppression of T cell proliferation in an IL-10-independent manner, thus amplifying the immunosuppressive effect by MSCs. This finding is interesting because it indicates a novel mechanism of IDO-mediated tolerance induction, mainly by inducing T-cell apoptosis/anergy and the generation of M2-type suppressor macrophages and regulatory T cells, in addition to biochemical mechanisms, including tryptophan depletion and several metabolites of the kynurenine pathway. In connection with such M2-type suppressor macrophages, the following situations are noteworthy. One pathway dependent on the TLR adaptor protein myeloid differentiation marker 88 (MyD88) induces the expression of arginase 1 during intracellular infections, whereas another pathway, which depends on the STAT6, is required for arginase 1 expression in M2 macrophages. Recently, Qualls et al. [53] reported that *M. bovis* BCG-infected macrophages produced soluble factors, including IL-6, IL-10, and granulocyte colony-stimulating factor (G-CSF), that induced the expression of arginase 1 characteristic of M2 alternatively activated macrophages in an autocrine-paracrine manner. Arginase 1 expression was controlled by the MyD88-dependent production of these cytokines rather than by cell-intrinsic MyD88 signaling to arginase 1. They revealed that the MyD88-dependent pathway that induced the expression of arginase 1 after infection by mycobacteria required STAT3 activation and that this pathway may cause the development of an immunosuppressive niche in granulomas because of the induced production of arginase 1 in surrounding uninfected macrophages. However, tyrosine phosphorylation of STAT-6, which is necessary for the expression of arginase 1 in response to IL-4, IL-13, or both, was not observed in this experimental system. Therefore, although BCG infection induces the expression of arginase 1 in macrophages, it is unlikely that these cells correspond to M2 alternatively activated macrophages.

 It is noteworthy that M2 macrophage subpopulations share functional properties characteristic of suppressor macrophages. Indeed, immature myeloid suppressor cells are known to have functional properties and a transcriptional profile related to M2 macrophages [22]. However, it may be noteworthy that M1 classically activated macrophages have also been demonstrated to display suppressor activity against lymphocytes by releasing immunosuppressive mediators including RNIs, TGF-*β* and prostaglandin E_2_ (PGE_2_). Thus, it is unclear whether such types can be regarded as “suppressor macrophages” and, indeed, some investigators argued that nitric oxide-mediated inhibition of the cytotoxic T lymphocyte (CTL) response by tumor-associated macrophages is merely a side effect of the activation of macrophages rather than a result of the action of a distinct subset of what have been termed suppressor macrophages [54]. Nevertheless, such macrophage populations are thought to play critical roles in the negative regulation of host protective immunity against tumors and microbial infections.

## 3. Immunosuppressive Functions of Alveolar Macrophages

In the tissues of the lungs and respiratory tract, where host immune cells have abundant opportunities to encounter and interact with inhaled antigens/immunogens and irritating substances/particulates of external origin, cellular functions of local T lymphocytes tend to be excessively upregulated in response to constitutive and potent antigenic signals. Therefore, negative immunoregulatory systems exist to maintain homeostasis in the lungs. Alveolar macrophages, as resident cells of the lungs, play critical roles in such systems. They have a distinct phenotype compared with other types of resident macrophages in the body. For instance, alveolar macrophages constitutively secrete proinflammatory cytokines, presumably as a result of stimulation by external particulates via their pattern-recognition receptors, including mannose receptors, scavenger receptors, and *β*-glucan receptors [55]. Thus, alveolar macrophages are central to innate defense systems of the airway. Moreover, they are also known to secrete immunosuppressive factors and act as immunoregulatory cells in the lungs. In fact, resident alveolar macrophages exhibit suppressive activity against the mitogen-induced proliferative response of T cells and antigen-presenting activity of dendritic cells. It thus appears that alveolar macrophages participate in the immunoregulation of T cell- and B cell-dependent immune responses in pulmonary tissues and milieus. Spiteri and Poulter [56] indicated that there are two subpopulations of human alveolar macrophages. One subset is immature-type macrophages with weak adherence to plastic plates, poor phagocytic capacity, and poor expression of the Fc receptor (FcR) and C3b receptor (CR3), but with strong functional activity as antigen-presenting cells and T-cell stimulator cells in allogeneic mixed lymphocyte reactions (MLRs). The other subset is mature-type macrophages with strong adherence, marked phagocytic ability and strong expression of FcR and CR3, but with poor activity in stimulating MLR. Notably, the latter FcR^high^ and CR3^high^ alveolar macrophage population acts as suppressor cells by repressing the MLR-stimulatory activity of the former FcR^low^ and CR3^low^ population.

 Furthermore, according to Upham et al. [57], human alveolar macrophages induced a reversible suppression of T-cell response to house dust antigens and phytohemagglutinin (PHA) without affecting profiles of CD3, CD2, CD28, and IL-2 receptor (IL-2R) expression and without reducing IL-2 production by target T cells, whereas such macrophages partly inhibited the secretion of IFN-*γ* by T cells. In this case, alveolar macrophages were found to markedly suppress the tyrosine phosphorylation of certain proteins involved in IL-2R-associated signaling pathways of T lymphocytes. Notably, the expression of such inhibitory activity by alveolar macrophages is achieved via heterogeneous mechanisms, involving both cell-to-cell contact with the target T cells and macrophage-derived humoral mediators including RNI and TNF-*α*. A similar finding was also reported for alveolar macrophages of guinea pigs, except that neither RNI nor PGE_2_ plays a critical role as a mediator of their suppressor action [58]. In any case, these findings may indicate that the immunoregulatory properties of alveolar macrophages are relatively selective, allowing T-cell activation and cytokine secretion while inhibiting T-cell proliferation within the lungs. Previously, Rich et al. indicated that the inhibitory activity of mouse alveolar macrophages against PHA-induced T-cell proliferation required cell-to-cell contact with target T cells and that the membranous phosphatidylglycerol of alveolar macrophages played important roles in their suppressor activity [59]. On the other hand, in the case of rat alveolar macrophages, cell-to-cell contact with target T lymphocytes is required for production of RNIs as a suppressor mediator, which inhibits concanavalin A (Con A)-induced T-cell mitogenesis [60]. It has also been reported that murine alveolar macrophages exhibited suppressor activity against IgM, IgG, IgA, and IgE antibody production by splenocytes [61]. In this case, it is unlikely that RNIs and ROIs participate in the suppressor function of the alveolar macrophages.

 Alveolar macrophages are composed of heterogeneous subpopulations of mononuclear phagocyte lineages with different phenotypes and functional properties. In this context, it has been demonstrated that resident alveolar macrophages could be activated by treatment with lymphokines of Con A-stimulated T cell origin in terms of elevated production of IL-1, IL-6, IL-12, TNF-*α*, and defects in TGF-*β* expression [62]. In contrast, the treatment of resident alveolar macrophages with 1-methyladenosine, an immunosuppressive molecule in tumor ascites fluids, caused the generation of a macrophage subpopulation possessing functional properties characteristic of suppressor macrophages, as follows: marked TGF-*β*-producing ability, low IL-6 expression, and defects in IL-1, IL-12, and TNF-*α* production [62]. Taken together, it can be concluded that some populations of alveolar macrophages participate in the immunological homeostasis of the lungs as negative immunoregulatory suppressors. With respect to the generation of immunosuppressive macrophage population in the lungs, the following findings by Arikawa et al. [63] concerning galectin-9, a *β*-galactoside binding lectin functioning as a ligand for T cell immunoglobulin- and mucin domain-containing molecule 3 (Tim-3), which is expressed on Th1 and Th17 cells, may be noteworthy. They found that galectin-9 was expressed on innate immune cells, such as dendritic cells, and expanded macrophages in bronchial lavage fluid to CD11b^+^Ly-6C^high^F4/80^+^ cells having immunosuppressive activity against T-cell proliferation [63]. This indicates that galectin-9 expands immunosuppressive macrophages in the lungs to ameliorate Th1/Th17 cell-mediated hypersensitive pneumonitis *in vivo*.

## 4. Immunosuppressive Macrophages Generated by Protozoal and Helminth Infections

Impairments of T-cell functions, such as proliferative responses to antigens and mitogens, and T cell-mediated immune reactions such as delayed-type hypersensitivity are frequently encountered during primary infections with protozoal organisms (*Trypanosoma*, *Toxoplasma*, etc.) and helminths (*Fasciola*, *Schistosoma*, etc.) [64–68]. It is generally recognized that the establishment of such immune unresponsiveness is mediated by suppressor macrophage populations generated 2 to 4 weeks after an infection. Suppressor macrophages induced by *Trypanosoma congolense* infection in mice suppressed the proliferative response of Con A-stimulated T cells via ROI- and prostaglandin-independent mechanisms [64]. On the other hand, murine splenic macrophages produced in response to African trypanosome (*Trypanosoma brucei rhodesiense*) infection exerted RNI- and prostaglandin-dependent suppressor activity against the proliferative response of Con A- and anti-CD3 antibody-stimulated T cells [65]. In this case, the generation of suppressor macrophages was in part dependent on IFN-*γ* and TNF-*α*. In particular, the combined effect of IFN-*γ* with certain soluble trypanosome products is crucial for the induction of suppressor macrophages characterized by enhanced RNI-producing ability due to an increase in the expression of iNOS [66]. *In vivo*, the generation of similar types of suppressor macrophages has been indicated in the cases of *Toxoplasma gondii* infection [67]. In this case, the cooperation of IL-10 and RNIs was found to be critical for the immunosuppressive action of suppressor macrophages against T-cell proliferation responding to mitogens, super antigens and parasite antigens. On the other hand, the generation of different types of suppressor macrophages has been reported in cases of filarial and theilerial infections [69, 70]. The suppressor macrophages induced in mice due to infection by filarial nematode, *Brugia malayi*, exerted their suppressive activity against lymphocyte proliferation in a fashion independent of RNIs, ROIs, and prostaglandins [69]. In *Theileria annulata*-infected cattles, two types of suppressor macrophages were induced. While the first-type macrophages expressed suppressor activity via a prostaglandin-mediated pathway, the second-type macrophages acted in a prostaglandin-independent manner [70].

 Chronicity, immune suppression, and Th2-type immune responses are characteristic features of infections with multicellular parasites [68]. Immune suppression and Th2-type responses have been attributed to chronic helminthic infections. In cases of helminth infections, the establishment of macrophage populations having suppressor activity against T-cell functions has been reported [71–73]. Loss of T lymphocyte proliferation concomitant with the emergence of a host response that is dominated by a Th2-type profile is well-established features of human filariasis. MacDonald et al. [71] reported that *Brugia malayi *infection in mice generated suppressor macrophage populations by an IL-4-dependent mechanism. The suppressor activity of these macrophages was partly dependent on IL-10. However, since T-cell suppression was induced by *B. malayi* even in the case of IL-10-deficient mice, IL-10 appears not to be essential for T-cell hyporesponsiveness induced by the filarial infection. It has been indicated that a *Schistosoma mansonii*-derived pentasaccharide, Lacto-*N*-fucopentaose (LNFP), induces suppressor macrophage populations with a Gr1^+^, F4/80^+^/CD11b^+^ (macrophage markers) phenotype via T cell-independent mechanisms, because such immunosuppressive macrophages could be generated in T-cell deficient SCID mice [72]. This type of suppressor macrophage blocked the anti-CD3- and anti-CD28-induced proliferation of naive CD4^+^ T cells through nitric oxide- and IFN-*γ*-dependent mechanisms [72]. It has recently been reported that immune suppression was induced in rats with advanced chronic fascioliasis in connection with the deviation to a Th2-type immune response [73]. In this case, mononuclear cell proliferation in the host spleen in response to T and B cell mitogens was strongly inhibited in infected rats. Notably, early in the infection, a Th2-type response predominated. However, this decreased in advanced chronic infection followed by the subsequent establishment of persistent immune suppression. It appears that the persistent immunosuppressed state characteristic of the advanced stages of fascioliasis is also mediated by certain suppressor macrophage populations generated responding to Th2-type immune deviation in infected hosts. In this context, a recent finding reported by Potian et al. [74] is interesting. They found that mice infected with the intestinal helminth *Nippostrongylus brasiliensis *(Nb) exhibited transitory impairment of resistance to coinfection with MTB. In their experimental model, although Nb infection induced a Th2 response in host mice, thereby resulting in the accumulation of M2 alternatively activated macrophages in the lung, the helminth-induced Th2 environment did not impair the onset of the MTB-specific Th1 immune response. Coinfected mice lacking IL-4R*α* exhibited improved ability to control MTB infection, which was accompanied by significantly reduced accumulation of M2 macrophages, suggesting the direct contribution of the IL-4R pathway to the heightened MTB susceptibility of coinfected mice. These findings indicate that the Th2 response can enhance the intracellular persistence of MTB, in part by mediating the alternative activation of M2-type suppressor macrophages via the IL-4R*α* signaling pathway.

## 5. Suppressor Macrophages Generated by Mycobacterial Infections

In hosts with mycobacterial infections, Th1-mediated immune responses are dominant and play crucial roles in the establishment and expression of antimycobacterial resistance. Figure 1 illustrates a cytokine network in hosts with mycobacterial infections. This network is composed of very complicated events mediated by various immunocompetent cells and a number of cytokines produced by these cells, including the following [11, 12, 75–79]: (1) activation/maturation of Th1 cells and NK cells in response to stimulatory signals due to proinflammatory cytokines, including IL-12, IL-23, IL-18, IL-27, TNF-*α*, IL-1, IL-7, and IL-15, which are produced by macrophages and dendritic cells stimulated with certain bacterial components of mycobacterial organisms; (2) activation of macrophages in response to activating signals by proinflammatory cytokines, such as IFN-*γ*, TNF-*α*, and GM-CSF produced by Th1 cells and NK cells. These immunological events mediated by the above cytokines are important for the establishment of mycobacterial immunity and the expression of host resistance. In contrast to the Th1/NK cell-mediated upregulation of macrophage antimycobacterial functions, the following findings have been obtained with respect to the roles of Th2 cytokines and other immunosuppressive cytokines in host resistance to mycobacterial infections [11, 12, 75–79]. First, IL-4 produced by Th2 cells, NKT cells, CD19^+^/B220^+^ B cells and neutrophils and IL-10 released from Th2 cells and macrophages down-regulate the maturation/activation of Th0 cells to Th1 cells directly or indirectly by inhibiting the production of IL-12 by macrophages. Second, Th1 cytokines (IFN-*γ*, etc.) and Th2 cytokines (IL-4, IL-10, etc.) mutually downregulate the activation of Th2 cells and Th1 cells, respectively. Third, immunosuppressive cytokines (IL-10, IL-13, TGF-*β*, etc.) produced by Th2 cells and infected macrophages act on macrophages in an autocrine or paracrine fashion, and thereby down-regulate the production of RNIs and ROIs and responsiveness to macrophage-activating cytokines such as TNF-*α* and IFN-*γ* [12, 76, 80]. In addition, It has recently been reported that blood levels of IL-9, which is presumably produced by Th2 cells, was elevated in TB patients compared with persons with latent MTB infection and that IL-9 reduced IFN-*γ* mRNA expression in peripheral blood mononuclear cells because of inhibition of Th1 cell differentiation [81]. These events lead to suppression of the bactericidal/bacteriostatic activity of host macrophages against mycobacterial pathogens.

 Mycobacteria cause severely depressed cellular immunity in the advanced stages of infection [82]. During the course of persistent and progressing mycobacteriosis in humans and experimental animals, the generation of immunosuppressive macrophages is frequently encountered [83, 84]. In the peripheral blood mononuclear cells of TB patients showing low tuberculin responses (anergy), the generation of suppressor macrophages populations, which markedly inhibit host T-cell proliferative responses to antigenic stimulatory signals with tuberculin-purified proteins, have been reported [83]. In addition, suppressor macrophage populations were generated among the spleen cells obtained from mice infected with BCG but not those from mice given heat-killed BCG [84]. The suppressor macrophages inhibited allogeneic mixed lymphocyte reaction and Con A-induced mitogenesis of T cells. In the cytokine network illustrated in Figure 1, Th2 cells produce IL-4 and IL-10, which are able to induce M2a-type alternatively activated macrophages, in response to IL-4/IL-1 signals [21]. In addition, macrophages, which have been primed with Th1 cytokines (IFN-*γ*) and other proinflammatory cytokines (GM-CSF, TNF-*α*, etc.) generated by NK cells and macrophages, produce IL-10 during infections with mycobacterial organisms. Notably, IL-10 is a potent generator of M2c-type immunoregulatory macrophages [17, 21]. Thus, these M2a and M2c macrophages seem to act as suppressor cells by producing IL-10 and TGF-*β* which downregulate T cell and macrophage functions [80, 85, 86]. On the other hand, certain subpopulations of classically activated M1 macrophages are endowed with suppressor cell activity against T lymphocytes, since such macrophages are capable of secreting RNI molecules and PGE_2_, both of which are potent suppressors of T-cell proliferative responses to antigenic and mitogenic signals and other T-cell functions, as described below. Therefore, it is thought that suppressor macrophage populations may be generated in hosts in the late phase of persistent and progressive mycobacterial infections.

### 5.1. Generation of Suppressor Macrophages during MAC Infections

 In studies of the present author for more than ten years, similar types of suppressor macrophages were found to develop in mice infected with MAC, as follows [87, 88]. (1) Splenic T-cell proliferative responses to the Con A stimulatory signal were severely reduced around 2 to 3 weeks after a bacterial challenge with a large inoculum of MAC pathogens, followed by a prolonged reduction in the responsiveness of T cells to Con A. (2) The generation of suppressor macrophage populations (plastic- and Sephadex G-10 column-adherent, Thy-1,2^−^cells) in the spleen of host mice was observed around the same periods after MAC infection and this was accompanied by the concomitant generation of splenic macrophage populations, which were characterized by a strongly increased ROI-producing ability in response to phorbol myristate acetate (PMA) triggering. (3) The bacterial elimination from the host spleen was most marked around weeks 2 to 3, indicating that the anti-MAC antimicrobial activity of splenic macrophages was most potently increased during the same periods after the MAC infection. These findings suggest that MAC-induced suppressor macrophages may simply correspond to a macrophage population, which acquired cellular functions characteristic of immunologically activated macrophages, that is, classically activated M1-type macrophages. Therefore, it is possible that the same mechanisms underlie the activation and acquisition of suppressor macrophage functions.

 In this context, for various types of peritoneal macrophages, including resident macrophages, macrophages induced with either thioglycollate, zymosan A, or a streptococcal cell wall preparation, and macrophages induced by BCG or MAC infection, there was a statistically significant correlation between their suppressor activity and ability to produce ROIs (*r* = 0.84, *P* < 0.005) [89]. Therefore, it is thought that the suppressor activity of a given macrophage generally correlates with its degree of activation in terms of ROI-producing ability. However, this relationship was not so tight. There were two exceptional populations, which were represented by points that deviate markedly from the normal bivariate distribution in the scatter diagram. That is, these macrophages had much greater suppressor activity than expected from the intensity with which they generated ROIs. Therefore, in certain types of macrophages there may be a dissociation between the ROI-producing ability and suppressor activity. These findings are consistent with another finding that the suppressor activity of test macrophages was not mediated by ROIs themselves. Similar findings on a dissociation between functions characteristic of an activated state and suppressor activity were reported by Boraschi et al. [90]; that is, macrophages activated by IFN-*γ* *in vitro* showed reduced suppressor activity against an antigen-specific lymphoproliferative response, although they did acquire marked tumoricidal capacity. On the other hand, such a reduction in suppressor activity was not noted when macrophages were activated by a lymphokine-rich supernatant of BCG-primed and purified protein derivative (PPD)-activated splenic T cells containing macrophage activating factor consisting of IFN-*γ* and other cytokines such as GM-CSF and TNF-*α*. Taken together, suppressor macrophages induced by mycobacterial infections may be composed of heterogeneous macrophage subpopulations consisting of at least the two types of macrophages described above.

### 5.2. Mechanisms of MAC Infection-Mediated Generation of Suppressor Macrophages

In order to assess the role of T cell-mediated immunity in the generation of suppressor macrophages during MAC infections, the profile of suppressor macrophages among host spleen cells in MAC-infected athymic nude mice was compared with that in MAC-infected euthymic mice. The following findings were made [91]. First, splenic macrophages possessing suppressor activity occurred not only in euthymic mice but also in athymic mice at around weeks 2 to 3. This implies that mature T cells are not a prerequisite for the generation of MAC-induced suppressor macrophage populations and that suppressor macrophage populations were produced not only through a T cell-dependent pathway but also through a T cell-independent mechanism in host animals with severe MAC infections. However, the suppressive activity was about four times greater in euthymic mice than in athymic mice, indicating that mature T cells are required for the generation of macrophage populations with highly potentiated immunosuppressive functions. In this context, it is noteworthy that PMA-triggered chemiluminescence, a parameter of macrophage activation on the basis of ROI-producing ability, was about twice as strongly increased in euthymic splenic macrophages than athymic splenic macrophage due to MAC infection. Therefore, MAC-induced splenic macrophages of both strains of mice were functionally activated in terms of an increase in PMA-responsiveness for a respiratory burst, although the activation was less extensive in athymic mice than in euthymic mice. Second, anti-TNF-*α*, anti-IFN-*γ*, and anti-TGF-*β* antibodies (Abs) but not anti-IL-6 Ab inhibited the MAC-induced generation of suppressor macrophages *in vivo*, and the neutralizing efficacy was in the order of anti-IFN-*γ* Ab > anti-TNF-*α* Ab > anti-TGF-*β* Ab [88]. In addition, the treatment of normal macrophages with either TNF-*α* plus IL-1*α* or TNF-*α* plus IFN-*γ* yielded a marked increase in the suppressor activity, with IL-1*α* plus IFN-*γ* having less of an effect [88]. These findings indicate the important roles of TNF-*α*, IFN-*γ*, and IL-1*α* in the MAC-induced generation of suppressor macrophages. Notably, TNF-*α* plus IFN-*γ* was the most active combination of cytokines tested, implying that T cell- and NK cell-derived IFN-*γ* plays an important role in the development of suppressor macrophages in hosts with MAC infections in addition to monokines such as TNF-*α* and IL-1*α*. In this context, a recent finding by Tatano et al. is interesting [92]. They examined profiles of the *M. intracellulare*-induced generation of immunosuppressive macrophages in MAC-susceptible BALB/c (*bcg^s^*) and resistant CBA/JN (*bcg^r^*) mice. They found that MAC infection in BALB/c mice caused the more rapid generation of immunosuppressive macrophages than MAC infection induced in CBA/JN mice. The suppressor macrophage population expressing macrophage markers, such as CD11b and F4/80, exhibited an increased ability to generate ROIs, and inhibited IL-2R expression by mitogenic T cells [92]. Thus, the *bcg* gene may be related to the generation of immunosuppressive macrophages in host mice.

 In the case of *Mycobacterium lepraemurium* infection in mice, similar types of mechanisms for the production of suppressor macrophages have also been reported [93–95]. The progressive impairment of cell-mediated immune functions in *M. lepraemurium*-infected mice has been attributed to the emergence of suppressor cells belonging to macrophage lineages. Gosselin et al. indicated that suppressor precursor cells were generated in spleen cells of *M. lepraemurium*-infected hosts harvested between 9 and 17 weeks after infection. The suppressor precursor cells (Fc*γ*R^+^CD11b^+^Ia^+^IgG^−^asialo-GM1^−^ adherent cells) were matured and developed suppressor activity against Con A-induced T-cell mitogenesis in response to stimulatory signals given by cell-to-cell contact (presumably involving a receptor-ligand type interaction) with nonadherent cells (Fc*γ*R^+^CD11b^+^Ia^−^Thy-1^−^CD4^−^CD8^−^IgG^−^asialo-GM1^−^ cells), which were distinct from mature T, B, and NK cells *in vitro* [93]. In this case, protein synthesis by the nonadherent regulatory cells was needed to exert their activity to cause maturation of the precursor suppressor macrophages [94]. The resulting suppressor macrophages depressed the Con A-mitogenic response of T cells through the inhibition of IL-2 production and expression of high-affinity IL-2R [95]. The expression of the suppressor macrophage activity was at least partly mediated by IFN-*γ* and prostaglandins. It should be noted that, in the case of *M. lepraemurium* infection, IFN-*γ* is not needed for the generation of suppressor macrophages *in vitro*, although this cytokine plays a critical role in the *in vitro* and *in vivo* induction of suppressor macrophages in MAC-infected mice. Therefore, it appears that there may be various mechanisms for the development of suppressor macrophage populations during the course of mycobacterial infections, depending on the mycobacterial species as an etiological agent, phase of infection, bacterial dose, and so forth.

### 5.3. Mechanisms for the Expression of Suppressor Activity by MAC-Induced Immunosuppressive Macrophages and Those Induced by MTB Infection

A series of studies on immunological mechanisms for the suppressor activity of immunosuppressive macrophages generated by MAC infection (MAC-induced suppressor macrophages) revealed the following. First, MAC-induced suppressor macrophages markedly inhibited the expression of IL-2R by Con A-stimulated T cells, while only moderately reducing IL-2-producing ability of T cells [87, 89, 91]. It thus appears that the major target of the MAC-induced suppressor macrophages is in the T-cell activation process acquiring IL-2 responsiveness through upregulation of IL-2R expression in response to T cell stimulating signals. Second, when either anti-TNF-*α*, anti-TGF-*β*, or anti-IFN-*γ* Ab was added to the culture medium, suppressor activity was markedly reduced, in the order of anti-TNF-*α*, anti-IFN-*γ*, and anti-TGF-*β* Abs [96]. By contrast, neither anti-IL-6 nor anti-IL-10 Ab exerted such a blocking effect. Therefore, TNF-*α*, IFN-*γ*, and TGF-*β* seem to be related to the full display of the suppressor function of MAC-induced suppressor macrophages. However, TNF-*α* and IFN-*γ* but not TGF-*β* were substantially lacking in inhibitory action against Con A-stimulated T-cell mitogenesis, when added exogenously. Hence, it is unlikely that TNF-*α* and IFN-*γ* directly modulated the proliferative response of T cells. On the other hand, both TNF-*α* and IFN-*γ* potentiated the effector function of the suppressor macrophages, whereas TGF-*β* acted to block the suppressor activity of MAC-induced immunosuppressive macrophages. In this context, when splenocytes harvested from MAC-infected mice were stimulated with Con A, membrane-bound TNF-*α* molecules were strongly expressed by MAC-induced splenic macrophages and large amounts of IFN-*γ* were secreted from MAC-induced splenic T cells [96]. Therefore, both TNF-*α* and IFN-*γ* produced by the MAC-induced suppressor macrophages themselves and MAC-sensitized T cells, respectively, act as the major regulatory cytokines that up-regulate the suppressor activity of MAC-induced macrophages in an autocrine or paracrine fashion. Third, since the suppressor activity of MAC-induced suppressor macrophages was severely blocked by *N^G^*-monomethyl-L-arginine (NMMA) and aminoguanidine (NOS inhibitors), an RNI-dependent mechanism is important for the expression of the immunosuppressive function of MAC-induced suppressor macrophages [96–98]. Indeed, this concept was supported by the finding that NOR 4 (nitric oxide donor)-derived RNIs actually inhibited Con A-induced T-cell mitogenesis [98]. Fourth, it is thought that other kinds of mediators, including PGE_2_ and free fatty acids, such as oleic acid and arachidonic acid, participate in the suppressor functions of the MAC-induced immunosuppressive macrophages for the following reasons [97, 98]. The suppressive activity of the suppressor macrophages was partly but significantly blocked by both indomethacin and quinacrine [98]. Moreover, both PGE_2_ and oleic acid actually suppressed Con A-induced T-cell proliferation. Phosphatidylserine (PS) was also found to exhibit strong inhibitory activity against T-cell mitogenesis, suggesting that it may also act as a mediator of the MAC-induced suppressor macrophages [98].

 It has been found that MAC-induced suppressor macrophages also exhibit inhibitory activity against LPS-induced B-cell mitogenesis [99]. While NMMA and Carboxy-PTIO (nitric oxide scavenger) effectively blocked the macrophage suppressor activity against Con A-induced T-cell mitogenesis, the suppressor action against B-cell mitogenesis was only weakly affected by these nitric oxide-reducing agents. Notably, B-cell mitogenesis was remarkably more susceptible to RNIs than T-cell mitogenesis. In addition, B-cell mitogenesis was less susceptible to the inhibitory effects of the other suppressor macrophage-derived mediators, including free fatty acids, TGF-*β* and PGE_2_, than T-cell mitogenesis. Therefore, there are significant differences in the modes of suppressor action of MAC-induced suppressor macrophages against T-cell and B-cell mitogenesis [99]. Alternatively, it is also possible that MAC-induced suppressor macrophage populations are composed of two subpopulations with distinct functional properties: M1-type suppressor macrophages which mainly suppress T-cell functions in an RNI-dependent manner and M2-type suppressor macrophages which mainly act on B cells by producing suppressor mediators other than RNIs, presumably IL-10, TGF-*β*, and so on.

 Because the suppressor macrophages are generated not only in MAC-infected mice but also MTB-infected mice, the profile of the generation and characteristics of suppressor macrophages during the course of MTB and MAC infections was investigated [88]. In both infections, a marked reduction in the Con A mitogenic response of splenic T cells was seen around 2 weeks after infection, and this was accompanied by the generation of potent immunosuppressive macrophages in the splenocytes of infected mice. The suppressive activity was much stronger in MTB-infected mice than in MAC-infected mice. In both infections, most of the suppressive macrophages exhibited suppressor activity that depended on the arachidonic acid cascade, particularly mediated by prostaglandins, and the remainder showed suppressor action independent of prostaglandins. The unique finding was that the generation of IL-2 reactive T-cell populations in splenocytes in response to the Con A signal was markedly inhibited by MAC- and MTB-induced immunosuppressive macrophages, whereas the suppressor macrophages failed to potently reduce the IL-2-producing ability of splenic T cells [88]. In this context, it has also been reported that the suppressor macrophages induced by filarial nematode, *Brugia malayi*, suppressed T-cell proliferation without causing the reduction of cytokine (IL-4) production by the target T cells [69]. In any case, these findings indicate a close similarity in immunosuppressive macrophages induced by MAC and MTB infections.

### 5.4. Mechanisms of Intercellular Transduction of the Suppressor Signals through Cell-to-Cell Contact from Suppressor Macrophages to Target T Cells

**Figure 2:**
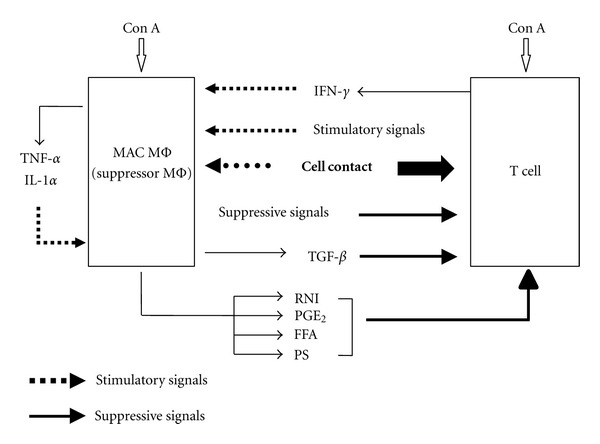
Mechanisms of intercellular transduction of immunosuppressive signals from MAC-induced suppressor macrophages to target T cells.

In the case of suppressor macrophages generated by *M. lepraemurium* infections, functional maturation of the suppressor precursor cells to acquire their suppressor activity was found to be dependent on cell-to-cell contact, presumably involving a receptor-ligand type interaction, with nonadherent regulatory cells [93]. Similarly, the inhibition of Con A-induced T-cell proliferation by MAC-induced suppressor macrophages was found to be dependent on cell contact of the suppressor macrophages with target T cells [98, 100]. That is, the expression of the suppressor activity of MAC-induced splenic macrophages was markedly reduced by separating target T cells from the macrophages using a Millipore filter in a dual chamber. In this case, the addition of mitomycin C-treated splenocytes to MAC-induced suppressor macrophages in the “bottom chamber," allowing cell-to-cell contact between the two, did not potentiate the humoral factor- (RNIs, PGE_2_, etc.) mediated expression of the suppressor activity of MAC-induced splenic macrophages through the Millipore filter. These findings indicate the following (Figure 2): (1) Intercellular transduction of suppressor signals from MAC-induced splenic macrophages to the target T cells is mediated by cell-to-cell contact between the two. (2) Cell contact between MAC-induced suppressor macrophages and splenocytes did not modulate the production of suppressor mediators such as RNI, free fatty acids (FFAs), phosphatidylserine (PS), PGE_2_, and TGF-*β* [96–98] by the macrophages themselves.

 As described above, PS exhibits strong inhibitory activity against T-cell mitogenesis, suggesting that it acts as a mediator of the MAC-induced suppressor macrophages [97, 98]. Notably, PS-mediated inhibitory activity against T-cell mitogenesis was not inhibited by quinacrine (phospholipase A_2_ inhibitor), thereby excluding the possibility that PS-derived free fatty acid moieties did not mediate the T cell-suppressing activity of PS [98]. In this context, it has been demonstrated that human alveolar macrophages exerted suppressor activity against PHA-induced T-cell mitogenesis through cell-to-cell contact with target lymphocytes [59, 101]. This inhibitory activity was partly attributable to a hydrophobic substance, which contained phosphatidylglycerol. Thus, it seems that intact PS and phosphatidylglycerol are needed for the suppressor activity. It is of interest that PS transported to the external leaflet of the plasma membrane acts as a membrane “flag” on apoptotic cells, resulting in the recognition and engulfment of these cells by phagocytes which possess PS receptors [102]. Therefore, the suppressor signal transmission from MAC-induced suppressor macrophages to target T cells via cell-to-cell contact seems to be partly mediated by PS molecules expressed on the surface of the macrophages.

 In addition, molecular biological studies on profiles of cell contact-mediated signal transduction from MAC-induced suppressor macrophages to the target T cells indicated the following [100, 103]. Ogasawara et al. [100] reported interesting findings as follows. First, the immunosuppressive macrophages displayed suppressor activity in an H-2 allele-unrestricted manner, indicating that MHC molecules are not required for cell contact. The macrophage suppressor activity was reduced markedly by paraformaldehyde fixation or treatment with cytochalasin B or colchicine, indicating that vital membrane functions are required for the immunosuppressive activity. Second, the suppressor activity of MAC-induced suppressor macrophages was independent of cell-to-cell interaction via CD40 ligand/CD40 and macrophage-derived indoleamine 2,3-dioxygenase, which causes rapid degradation of tryptophan in T cells. Third, precultivation of splenocytes with MAC-induced suppressor macrophages, allowing cell-to-cell contact, reduced Con A- or anti-CD3 antibody-induced mitogenesis but not PMA/calcium ionophore A23187-elicited proliferation of T cells. In addition, cocultivation of T cells with MAC-induced suppressor macrophages caused marked changes in profiles of the tyrosine phosphorylation of 33-kDa, 34-kDa, and 35-kDa proteins and, moreover, the activation of protein kinase C (PKC) and its translocation to the cell membrane. It thus appears that suppressor signals of MAC-induced macrophages, which are transmitted to the target T cells via cell contact, principally cross-talk with the early signaling events before the activation of PKC and/or intracellular calcium mobilization.

 Although the 33-, 34-, and 35-kDa proteins have not yet been identified, the following can be stated. First, these proteins are distinguished from Fyn, Lck, ZAP-70, Vav, Hs1, Cb1, SLP-76, Grb-2, LAT, SOS, and PI3K, which are known to play roles in the early stages of T-cell receptor (TCR) signaling [104, 105], on the basis of their molecular weights. Similarly, on the basis of molecular weight, the 33-kDa and 34-kDa proteins are also distinguished from Csk, SHP-1, gab2 and SHIP-1 that suppress TCR signaling pathways via inhibition of Fyn and Lck [104, 105]. Second, the 35-kDa protein may correspond to HS1-associating protein X-1 (HAX-1), which is directly associated with HS1, a substrate of the Src family and Syk/ZAP-70 tyrosine kinases that play important roles in the early events of TCR-mediated signaling in T cells [106, 107]. The 35-kDa protein may also correspond to a protein which is phosphorylated by a CD8-coupled protein-tyrosine kinase p56lck [108]. In any case, it is noteworthy that there is cross talk between suppressor macrophage-mediated suppressor signals, which are transmitted to target T cells via cell-to-cell contact, and TCR-associated signaling pathways, thereby causing the inhibition of tyrosine phosphorylation of certain proteins. In this context, a separate experiment showed that the reduction of cAMP levels in target T cells did not affect the MAC-induced suppressor macrophage-mediated suppression of Con A-induced T-cell mitogenesis [100]. It is thus unlikely that the suppressive signals from the suppressor macrophages cross-talk with the activation of cAMP-dependent protein kinase or its downstream events hindering T-cell activation processes, such as inhibition of TCR ligation-coupled Lck autophosphorylation and intervention in the activation of ERK and JNK [109, 110].

 Further investigations by Shimizu et al. [103] concerning mechanisms of signal transduction from MAC-induced suppressor macrophages to target T cells revealed the following. First, it was found that a novel B7-1-like molecule (B7-1LM) recognizable with one of three test clones of anti-B7-1 monoclonal Abs (mAbs) was required for expression of the macrophage suppressor activity. Neither anti-B7-2, anti-intercellular adhesion molecule-1 (ICAM-1), nor antivascular cell adhesion molecule-1 (VCAM-1) mAb blocked the macrophage suppressor activity. These findings suggest that transmission of the suppressor signals from MAC-induced suppressor macrophages to target T cells via cell contact was dependent on B7-1LM, which shares in part the same epitope with B7-1. This concept is further supported by the following findings [103]. (1) The expression of B7-1LM on MAC-induced splenic macrophages was correlated with their suppressor activity. (2) Cell-to-cell binding of MAC-induced suppressor macrophages with target T cells was inhibited by the anti-B7-1 mAb (clone 16-10A1). (3) The blocking of cytotoxic T-lymphocyte-associated protein 4 (CTLA-4) molecules on target T cells did not attenuate the macrophage suppressor activity, indicating that CTLA-4 does not act as a B7-1LM receptor, and that macrophage-derived suppressor signals are transmitted to target T cells through the interaction of B7-1LM with unknown putative receptor molecules other than CTLA-4 on the T cells. Separate experiments indicated that CD28 does not act as a B7-1LM receptor either. In any case, these findings indicate that a B7/CTLA-4-independent mechanism is needed for the transmission of the suppressor signals from MAC-induced suppressor macrophages to target T cells.

 Second, Con A stimulation of cellular functions of MAC-induced suppressor macrophages was needed for effective cell contact with target T cells and subsequent expression of the suppressor activity of the macrophages [103]. Notably, the Con A-induced increase in the suppressor activity of MAC-induced suppressor macrophages was not inhibited by herbimycin A, H-7, or H-88. The results obtained with these metabolic inhibitors suggest that Con A signal-associated expression of the suppressor activity of MAC-induced splenic macrophages does not involve signaling pathways which are mediated by protein tyrosine kinases (PTKs), PKCs, or cAMP-dependent protein kinases. On the other hand, the calcium/calmodulin-dependent protein kinase II (CaMKII) inhibitor KN-62 partially attenuated the suppressor activity of the macrophages, indicating that CaMKII-mediated signaling may play important roles in the activation of MAC-induced splenic macrophages in response to Con A in terms of acquisition of the suppressor activity. In this context, it is noteworthy that KN-62 has inhibitory activity against ATP/P2X_7_ receptors [111]. It has been reported that ATP-induced stimulation of P2X_7_ receptors on macrophages is associated with a marked increase in the activity of phospholipase D, causing a potentiation of the antimycobacterial activity of the macrophages [112]. Notably, it has been reported that ATP-induced microbicidal activity of macrophages is attenuated by KN-62 but not by inhibitors of PTK, PKC, and adenylate cyclase [111]. Therefore, it is possible that ATP/P2X_7_ interaction on MAC-induced splenic macrophages is needed for their suppressor activity against target T cells.

## 6. Concluding Remarks

Suppressor macrophage populations having extensive immunosuppressive activity against lymphocyte functions are generated during the course of mycobacterial infections, particularly in hosts with severe infections. There have been reports of the generation of such suppressor macrophages in cases of human or mouse infections with MTB, *M. bovis* BCG, MAC, and *M. lepraemurium*. Suppressor macrophages are also likely to be generated by infections of pathogenic nontuberculous mycobacteria other than MAC, such as *M. kansasii*, *M. marinum*, *M. scrofulaceum*, *M. gordonae*, *M. ulcerans*, *M. fortuitum*, and *M. abscessus*. The generation of suppressor macrophages is closely related to the subsequent severe and persistent impairment of host T lymphocyte functions, such as antigen/mitogen-induced proliferative response, cytokine production, and cytotoxic capacity in a mixed lymphocyte reaction [83, 84]. It thus appears that suppressor macrophages participate in the abrogation of host immune resistance to mycobacterial pathogens in the advanced stages of infection. In particular, with respect to the suppressor macrophage populations induced by MAC infection, the following suggestions can be made.

 First, based on macrophage polarization, macrophages are composed of four subpopulations having functional properties characteristic of (1) M1-type classically activated (IFN-*γ*/TNF-*α*-induced) macrophages, (2) M2a-type alternatively activated (IL-4/IL-13-induced) macrophages, (3) M2b-type type II (immune complex- and TLR/IL1-R ligand-induced) macrophages, and (4) M2c-type deactivated (IL-10-induced) macrophages [17, 18]. Macrophages belonging to M2 types are known to produce large amounts of anti-inflammatory/immunomodulatory cytokines, IL-10 (M2a, M2b, M2c macrophages), and TGF-*β* (M2c macrophages) as central mediators of their immunosuppressive effects on T-cell functions [17, 18]. As reported by Schreiber et al. [16], MTB-induced excessive expression of IL-10 in macrophages promotes the M2 (M2c) polarization program displaying diminished antimycobacterial function of macrophages. Transgenic mice overexpressing IL-10 in a macrophage-specific fashion were indeed susceptible to MTB infection, displayed a specifically suppressed IL-12 in infected tissues, and were characterized by lung macrophages with an M2 phenotype enabling MTB infection. Taken together, it is thought most of suppressor macrophages generated due to heavy/advanced infection by mycobacterial pathogens may correspond to M2 macrophage populations.

 It is unknown why protective cytokines, such as IFN-*γ* and TNF-*α*, lead to the generation of immunosuppressive macrophage populations in the case of MAC infection. As described previously (Section 2), after MAC infection, the first responder M1 macrophages generated in response to the signaling of TNF-*α*, IL-1*β*, and IFN-*γ* usually exhibit an inflammatory phenotype and secrete proinflammatory/microbicidal mediators, such as RNIs and ROIs, thereby causing the activation of antimicrobial mechanisms characteristic of M1 classically activated macrophages. However, these radicals are toxic and highly damaging to neighboring tissues. Therefore, M1 macrophages must be controlled to prevent collateral extensive tissue damage by regulatory mechanisms, including the generation of M2 alternatively activated macrophages, which antagonize M1 macrophage polarization and also exhibit strong anti-inflammatory activity [19]. The resulting M2 macrophages are detected as suppressor macrophages induced due to MAC infection in mice (Section 5.2).

 In this context, the possibility cannot be excluded that M1 classically activated macrophages may also exhibit suppressor activity against T cell functions, since they secrete RNIs, which act as immunosuppressing effectors against T cell proliferation. Indeed, in the authors' experimental model, suppressor macrophages, which were induced by MAC infection, exerted their suppressor activity by producing RNI molecules, the production of which is a critical phenotype of M1 classically activated macrophages [17–19, 97, 98]. Indeed, this is also the case for suppressor macrophages generated in response to trypanosomal infections [66]. In addition, the immunosuppressive activity of MAC-induced suppressor macrophages is strongly correlated with their ability to produce ROIs, which are exclusively produced by M1 macrophages [17, 20, 21]. However, M2b macrophages (type II-activated macrophages/regulatory macrophages) characterized by low IL-12 and high IL-10 production have a phenotype of iNOS^high^, thereby having a potent activity to produce RNIs [29, 30, 113]. Therefore, it is also possible that M2b macrophages involved in the MAC-induced suppressor macrophage populations exhibited suppressor activity against T-cell functions instead of M1 macrophages. It is therefore necessary to determine whether such M1 macrophages possessing suppressor activity can be truly identified as suppressor macrophages, by performing further studies.

 Second, it can be concluded that the suppressive signals from MAC-induced suppressor macrophages are transmitted to target T cells through cell contact between a novel B7-like cell surface molecule on the macrophage and certain receptor(s) other than CTLA-4 and CD28 on target T cells (Figure 2) [103]. Moreover, it is thought that the suppressor signals of MAC-induced macrophages are at least partly transmitted to target T cells via cell-to-cell contact in addition to intercellular interaction between the suppressor macrophages and T cells using humoral suppressor mediators, such as RNIs, PGE_2_, free fatty acids (FFA), and TGF-*β*. Interestingly, the suppressor signals transmitted to target T cells by cell-to-cell contact are thought to cross talk with tyrosine phosphorylation-mediated signal transduction pathways in the TCR-associated signaling events in target T cells [100, 103]. It is of interest to know the precise roles of 33-kDa, 34-kDa and 35-kDa proteins, the tyrosine phosphorylation profiles of which are significantly affected due to the suppressor signals transmitted from MAC-induced suppressor macrophages to target T cells via cell contact, in the expression of inhibitory activity of the suppressor macrophages against T-cell functions. Further studies based on molecular biology and cell technology are desired to elucidate the precise mechanisms of the suppressor macrophage-mediated downregulation of T-cell functions.

 Third, with respect to variations in the properties of suppressor macrophage populations depending on the pathogen, the following conclusion may be possible, on the basis of the nature of extracellular mediator molecules in the expression of inhibitory activity against T-cell proliferation. The immunosuppressive macrophages induced by MAC exhibit their suppressor activity by producing RNIs and prostaglandins as immunosuppressive mediators [96–98]. This feature is common to those of suppressor macrophages induced by a protozoa *Trypanosoma brucei rhodesiense* [65, 66]. Although the suppressor activity of *Toxoplasma gondii*-induced macrophages is also dependent on RNIs, as in the case of MAC-induced suppressor macrophages, IL-10 plays important roles as a suppressor mediator in *T. gondii*-induced macrophages but not in MAC-induced macrophages [67]. Notably, *Brugia malayi*-induced suppressor macrophages exert suppressor activity using IL-10 as a mediator [71]. Moreover, although ROIs plays no significant roles as a mediator of *Trypanosoma congolense*-induced suppressor macrophages as in the case of MAC-induced suppressor macrophages, the latter but not the former macrophages use prostaglandins as a suppressor mediator [64, 98]. These situations may indicate that the common extracellular mediators of the suppressor macrophages induced by MAC and protozoal infections are all RNIs. In contrast, the modes of participation of other mediator molecules, such as prostaglandins and IL-10, in the manifestation of immunosuppressive activity of infection-induced suppressor macrophages diverse vary among pathogens.

 Fourth, it is of interest to know the roles of MAC-induced suppressor macrophages in the development or diminishment of the protective immunity of hosts against MAC infection. In mice given large challenge doses of MAC, suppressor macrophages were generated in the spleen of host mice during weeks 2 to 3 after infection, followed by subsequent reduction of the responsiveness of splenic T cells to Con A stimulation and TCR signaling induced by anti-CD3/anti-CD28 antibodies. This reduction was very severe and sustainable for long periods, especially in the case of MAC-susceptible BALB/c strain mice [92]. Therefore, MAC-induced suppressor macrophages are thought to cause long-term downregulation of T-cell functions in host animals, causing severe impairment of the onset of the T cell-mediated mycobacterial antigen-specific immune response and the establishment of prolonged reduction of protective immunity against MAC. Therefore, control of the generation of such suppressor macrophages may be useful for clinical control and vaccine-based prophylaxis of MAC diseases and presumably also TB.

 Finally, during the course of persistent and progressing TB in humans, the generation of immunosuppressive macrophages is frequently encountered. Indeed, in the peripheral blood mononuclear cells of TB patients showing low tuberculin responses (anergy), the generation of suppressor macrophages populations, which markedly inhibit host T-cell proliferative responses to antigenic stimulatory signals with tuberculin-purified proteins, have been observed by Ellner [83]. Although only one report has been published on the generation of a suppressor macrophage population in the case of human TB, it has been demonstrated that M2 polarization of host macrophages is actually encountered in human TB [15, 18, 45, 51]. As described above in detail, M2 alternatively activated macrophage populations essentially correspond to immunosuppressive macrophages. This may indicate that suppressor macrophages affect the profiles of anti-MTB immune response in TB patients, thereby causing significant modification of MTB-mediated pathogenesis. Therefore, it may be beneficial for clinical control of refractory mycobacteriosis to prevent the generation of suppressor macrophages, especially M2-type immunosuppressive macrophages, during the course of chronic infection. Since an excessive immune deviation to Th1 and M1 programs compensatorily causes the generation of M2-type suppressor macrophages because of homeostasis, it is reasonable to control Th1 and M1 activation so as not to reach excessive levels. This strategy may be achieved by using mildly acting anti-inflammatory drugs or antimycobacterial drugs, which exhibit not only antimicrobial activity but also mild anti-inflammatory and/or immunosuppressing effects. For instance, some traditional Chinese medicines having mild anti-inflammatory activity may be useful. For this purpose, plants belonging to the genus *Broussonetia*, which are used in traditional medicine in Asian countries such as China and Japan, are expected to be useful, since these plants exert mild anti-inflammatory activity [114]. Among the major constituents of the plants, papyriflavonol exhibit inhibitory effects on inflammatory secretory phospholipase A_2_ and broussochalcone and kazinol suppress RNI production [114]. In addition, macrolides are known to have immunomodulatory effects that are beneficial for patients suffering from chronic pulmonary inflammatory syndrome, such as diffuse panbronchiolitis, cystic fibrosis, asthma and bronchiectasis [115]. It is expected that future systematic studies will provide good evidence for this strategy. This strategy may be applicable to development of new useful vaccines against TB and MAC diseases.

 Overall, this paper has described the cellular characteristics of suppressor macrophages and, moreover, M2-type immunosuppressive macrophages induced by mycobacterial and protozoal infections. Unfortunately, essentially no reports have described the nature of suppressor (immunosuppressive) macrophages induced by general microbial infections, such as those due to common bacteria and fungi, other than mycobacterial infections (TB and MAC infection) and protozoal infections, as far as we have searched using internet document retrieval systems. Future studies on the suppressor macrophages generated in hosts suffering from infections with various types of bacterial infections will elucidate interesting features of suppressor macrophages, particularly the cellular mechanisms of their suppressor activity.

## Figures and Tables

**Table 1:** Functional profiles of M1, M2, and suppressor macrophages^a^.

Expression of	Degree of expression or production		
	M1 MΦs^b^	M2 MΦs	Suppressor MΦs^c^
IL-12	++	−	
IL-23	++	−	
IL-10	−	++	+
TNF-*α*	++	− (M2b+)^d^	+
IL-1	++	− (M2b+)^d^	
IL-1ra	+	++ (M2a, M2c)^e^	
IL-6	++	− (M2b+)^d^	++
Type I IFN	++	−	
TGF-*β*	−	+ (M2c)	−
CCL1	−	++ (M2b)	
CCL2, 5, 15, 19	+++	+ (M2a)	
CXCL9, 10, 11,16	+++	+	
CCL13, 17, 18, 22, 23, 24	+	++ (M2a)	
IL-1R1	++	+	
IL-2R*α*	++	+ (M2a)	
IL-15R*α*	++	+ (M2a)	
Scavenger receptor	−	+ (M2c)	
Mannose receptor	+	+++ (M2a, M2c)	
TLR2, TLR4	++	+	
TLR5	+	++ (M2a)	
CD14	+	++ (M2c)	
FcR^b^	++	+	
CCR2	+	++ (M2c)	
CCR7	+++	−	
CXCR4	+	++ (M2a)	
MHC-II	+	− (M2b+)^d^	
CD86 (B7.2)	+	− (M2b+)^d^	
Fizz1^b^	−	++ (M2a)	
Ym1^b^	−	++ (M2a)	
Galectin-3	+	++ (M2a, M2c)	
iNOS	++	− (M2b++)^d^	+
Arginase 1	−	++ (M2a, M2c)	
IPD^b^	+++	+	
COX-1^b^	+	++ (M2a)	
COX-2^b^	++	− (M2a)	
RNI	++	− (M2b+)^d^	++
ROI	++	− (M2b+)^d^	++
Polyamine	−	++ (M2a, M2c)	

^a^Previous findings described in the following papers are summarized: in References [18, 20, 21, 27, 29, 36–38].

^b^Abbreviations: MΦs, macrophages; FcR, Fc receptor; Fizz1, found in inflammatory zone 1; Ym1, M2-associated chitinase-like protein; IPD, indoleamine-pyrrole 2,3 dioxygenase; COX, cyclooxygenase.

^c^Findings on suppressor macrophages induced by mycobacteriosis and protozoiasis are indicated. In cases of these macrophages, profiles of cytokine, chemokine, receptor, and enzyme expression other than those indicated in this table have not yet been studied, as far as we know.

^d^Exceptionally positive in the case of M2b macrophages.

^e^ Positive or negative, especially in the cases of indicated macrophage populations.
